# Porous tantalum-composited gelatin nanoparticles hydrogel integrated with mesenchymal stem cell-derived endothelial cells to construct vascularized tissue *in vivo*

**DOI:** 10.1093/rb/rbab051

**Published:** 2021-09-16

**Authors:** Zhenhua Zhao, Mang Wang, Fei Shao, Ge Liu, Junlei Li, Xiaowei Wei, Xiuzhi Zhang, Jiahui Yang, Fang Cao, Qiushi Wang, Huanan Wang, Dewei Zhao

**Affiliations:** 1 Orthopaedic Department, Affiliated ZhongShan Hospital of Dalian University, No. 6 Jiefang Street, Zhongshan District, Dalian, Liaoning 116001, P. R. China; 2 National-Local Joint Engineering Laboratory for the Development of Orthopedic Implant Materials, Affiliated ZhongShan Hospital of Dalian University, No. 6 Jiefang Street, Zhongshan District, Dalian, Liaoning 116001, P. R. China; 3 Key State Laboratory of Fine Chemicals, School of Bioengineering, Dalian University of Technology, No. 2, Linggong Road, High-Tech District, Dalian 116024, P. R. China; 4 School of Mechanical Engineering, Dalian Jiaotong University, Dalian 116028, P. R. China; 5 Reproductive Medicine Centre, Affiliated ZhongShan Hospital of Dalian University, No. 6 Jiefang Street, Zhongshan District, Dalian, Liaoning 116001, P. R. China; 6 Department of Biomedical Engineering, Faculty of Electronic Information and Electronical Engineering, Dalian University of Technology, Dalian 116024, P. R. China; 7 Laboratory Animal Center, Affiliated ZhongShan Hospital of Dalian University, No. 6 Jiefang Street, Zhongshan District, Dalian, Liaoning 116001, P. R. China

**Keywords:** porous tantalum, gelatin nanoparticles hydrogel, bone marrow mesenchymal stem cell, endothelial cell, vascularization

## Abstract

The ideal scaffold material of angiogenesis should have mechanical strength and provide appropriate physiological microporous structures to mimic the extracellular matrix environment. In this study, we constructed an integrated three-dimensional scaffold material using porous tantalum (pTa), gelatin nanoparticles (GNPs) hydrogel, and seeded with bone marrow mesenchymal stem cells (BMSCs)-derived endothelial cells (ECs) for vascular tissue engineering. The characteristics and biocompatibility of pTa and GNPs hydrogel were evaluated by mechanical testing, scanning electron microscopy, cell counting kit, and live-cell assay. The BMSCs-derived ECs were identified by flow cytometry and angiogenesis assay. BMSCs-derived ECs were seeded on the pTa-GNPs hydrogel scaffold and implanted subcutaneously in nude mice. Four weeks after the operation, the scaffold material was evaluated by histomorphology. The superior biocompatible ability of pTa-GNPs hydrogel scaffold was observed. Our *in vivo* results suggested that 28 days after implantation, the formation of the stable capillary-like network in scaffold material could be promoted significantly. The novel, integrated pTa-GNPs hydrogel scaffold is biocompatible with the host, and exhibits biomechanical and angiogenic properties. Moreover, combined with BMSCs-derived ECs, it could construct vascular engineered tissue *in vivo*. This study may provide a basis for applying pTa in bone regeneration and autologous BMSCs in tissue-engineered vascular grafts.

## Introduction

Tissue engineering (TE) is expected to become a new approach to create alternative tissues for treating congenital deficiency or pathological tissues. For tissue regeneration, the key problem for TE is a vascular deficiency in the engineered structure [1]. Given the diffusion distance limitation, rebuilding the blood vessels' network is challenging to promote the supply of nutrients, gas exchange, and elimination of waste products [2, 3]. To overcome the problem of vascularization, many scholars have proposed numerous methods, such as embedding angiogenic factors into stents to advance the growth of microvessels, manufacturing techniques for developing polymers, including vascular-like networks, and vascularization of the matrix before cell inoculation [4]. A three-dimensional (3D) scaffold is a critical element in TE researches. It could provide the matrix for cellular anchorage, migration, and growth. In addition, it promotes neovascularization and tissues regeneration by supplying nutrients and oxygen. Furthermore, the fibrous porous structures of the 3D scaffold can mimic the extracellular matrix (ECM) environment and significantly improve cell functions, including angiogenesis [5, 6].

Tantalum (Ta) has received extensive attention in biomedicine due to its excellent biocompatibility and chemical stability [7]. Since 1940, Ta has been used in clinical practice and widely applied in diagnostics and implantation, such as radiographic markers, vascular clips, endovascular stents, cranioplasty plates, orthopedics, and dental implants [8]. Compared with traditional metal implant materials (e.g. stainless steel, titanium alloys and cobalt-based alloys), Ta has superior ductility, tenacity biocompatibility, and high corrosion resistance. Porous metal materials have attracted extensive attention among biomaterials. Porous tantalum (pTa) has been regarded as the ideal orthopedic implant material with promising compatibility and mechanical support [9]. Clinical studies have shown that pTa implant material has good biocompatibility and is integrated well with bone tissue. In addition, this combination has superior long-term stability and is not easy to loosen [10, 11]. Angiogenesis is the primary element for bone regeneration; intramembranous and intrachondral ossification occur around the growth of vascular tissues [1]. In bone regeneration, such as bone defects, the ideal scaffold should have fibrous porous structures in which the reconstruction of vascularization and the formation of new bone should be carried out [12]. However, pTa does not have the appropriate structure to mimic the ECM microstructure. Therefore, pTa is lacks of property to promote angiogenesis and limits its application in bone regeneration.

Recently, many investigations have made numerous attempts on vascularized structures *in vitro*. One method is to inoculate seed cells on the suitable scaffold with mechanical properties, stimulating rapid cell growth and directed differentiation *in vitro*. Therefore, when implanted *in vivo*, the designed structure will undergo reconstruction and maturation to repair or form functional tissue [13]. The inherent angiogenic ability of endothelial cells (ECs) can be used to avoid angiogenesis induced by prefabricated channels or growth factors. However, the clinical application of mature ECs from autologous vascular tissue has certain limitations; (i) The isolation process requires an invasive operation; (ii) the proliferation potential of mature ECs is relatively low; and (iii) to obtain sufficient cells from small quantities of autologous tissue biopsies is challenging. These limitations have prompted the search for ECs sources with more proliferative and angiogenic abilities. Bone marrow-derived hematopoietic stem cells (HSCs), endothelial progenitor cells, mononuclear cells, and mesenchymal stem cells (MSCs) can differentiate into ECs. Meanwhile, bone marrow mesenchymal stem cells (BMSCs) are pluripotent precursors that can differentiate into various cell types derived from mesoderm, including osteocytes, chondrocytes, adipocytes, and stromal cells [14]. Nevertheless, the angiogenic potential of BMSCs-derived ECs in 3D scaffold material *in vivo* is rarely reported.

Colloidal gels are a special class of hydrogel materials, showing various applications, including ceramic processing, food industry, and biomedical engineering. Previous studies confirm hydrogel provides a physiologically relevant microenvironment for cell and tissue regeneration [15, 16]. However, traditional permanent hydrogels with a wide range of high cross-linking density cannot adapt to the complexity of the local environment and bones’ irregular structures due to its elasticity being more advantageous than viscoelasticity [17]. Adaptable hydrogels can exhibit ideal viscoelasticity and withstand complex mechanical environments and irregular shapes because of the reversibility of physical network connections [18]. Previously we reported a novel class of colloidal gels assembled by gelatin nanoparticles (GNPs) of electrostatic attraction. These gels are composed of a porous particulate network dispersed in a continuous phase of an aqueous solvent. This particulate microstructure renders colloidal gels superior properties like tailorable viscoelasticity, incorporating other components, and fascinating mechanical behavior. The reversible feature of the interparticle forces renders the colloidal gels shear-thinning and self-healing, thereby enabling these gels to be injectable and moldable. More importantly, GNPs within the colloidal gels can sustain release therapeutics, including proteins or small molecule drugs, which are an attractive feature for regenerative medicine [19, 20]. However, adapting to the mechanical environment and local structure *in vivo* remains a challenge.

Hereby, to expand the application of pTa in bone regeneration, we constructed artificial cellular scaffolds that could mimic the microstructure of natural tissue for vascular TE application. In the present study, the combination of pTa and GNPs hydrogel was designed to construct the 3D scaffold material and expected to have the advantages of two materials with a particular mechanical strength for cell and tissue regeneration, providing physiological relevant microenvironment. The characteristics and biocompatibility of pTa and GNPs hydrogel scaffold were evaluated by mechanical testing, scanning electron microscopy (SEM), cell counting kit, and live-cell assay. Then, we proposed a pTa-GNPs hydrogel scaffold combined with differentiated endothelial BMSCs. Using *in vivo* animal study, we determined whether the BMSCs-derived ECs could promote angiogenesis in the scaffold.

## Materials and methods

### Ta samples preparation and morphological analysis

**Figure 1.**
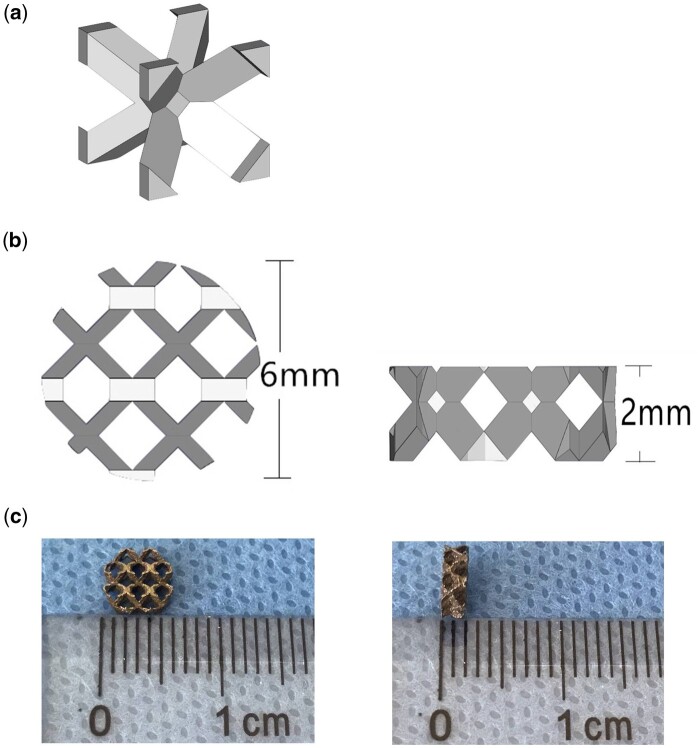
Design of pTa sample. (**a**) The body-centered cubic unit cell. (**b**) CAD model of disc-shaped pTa. (**c**) Disc-shaped pTa used in biological experiment (*φ* 6 mm×*H* 2 mm).

The columnar and disc-shaped structures of pTa samples were prepared by computer-aided design software. The cell structure (body-centered cubic structure; Fig. 1a) was imported in the .stl file, and the sample model was generated by computer-aided design (CAD) software. Porous Ta samples were created by selected laser melting (SLM; AM400, Renishaw, Britain) based on predesigned CAD data. The laser power and laser scanning speed were set at 320 W and 500 mm/s. Meanwhile, the Ta powder layer thickness and the hatching distance were 30 and 70 μm, respectively. The pTa cylinders (*φ* 6 mm × *H* 10 mm) and pTa discs (*φ* 6 mm × *H* 2 mm; Fig. 1b and c) were used in mechanical testing and biological experiment, respectively. The disc samples were autoclaved at 121°C for 30 min to prepare for cytotoxicity and *in vivo* experiments. For the design, the porosity, strut size, and pore diameter of pTa were 88.6%, 430μm, and 1300 μm, respectively. The gravimetric method was used to calculate the porosity of pTa cylindrical samples [21]. A SEM (SU3500, Hitachi, Japan) was used to examine the sizes and morphology of the sample, and Image J 1.8 (National Institutes of Health, USA) was used to analyze data.

### Mechanical testing

The mechanical properties of five cylindrical pTa (*φ* 6 mm × 10 mm) were evaluated by compression test. The universal testing machine (ETM 503A, Wance, China) was used to conduct mechanical tests, and the constant deformation rate was 1 mm/min. The compression elastic modulus was analyzed by the linear region of the stress–strain curve.

The rheological properties of the GNPs were measured by a rheometer (DHR, TA Instruments, USA). All measurements were performed using a flat plate with a diameter of 20 mm at 25°C with a gap distance of 800 µm. First, the storage modulus *G′* and loss modulus *G″* were determined using an oscillatory time sweep test for 300 s at a constant strain of 1 0.5% and a constant frequency of 1 Hz. Subsequently, a frequency sweep measurement was performed at a constant strain of 0.5% and the angular frequency range from 0.628 to 628.319 rad/s. To measure the viscosity, these gels were loaded with steady rate sweeps within a shear rate range of 0.1–100 s^−^^1^. Self-healing properties of the sample were quantitatively characterized by monitoring the evolution of storage (*G″*) and loss (*G″*) modulus of gels during multiple cycles of destructive shearing (oscillatory strain sweep with increasing strain from 0.1% to 500% with a fixed frequency of 1 Hz) and recovery (oscillatory time sweep at 0.5% strain and a frequency of 1 Hz) for 200 s.

### Preparation of GNPs

The method of two-step desolvation was carried out to prepare GNPs [5]. Briefly, 1 g gelatin was dissolved in 20 ml of distilled water with constant heating and added 25 ml of acetone to precipitate the gelatin chains of high molecular weight and obtain the gelatin solution (5% w/v). The gelatin became soluble in water at 40°C after removing the supernatant, and then the pH of the gelatin solution was adjusted to 2.5. Subsequently, the GNPs were formed after adding 75 ml of acetone to the gelatin solution with vigorously stirring (1000 rpm) at a constant rate of 3.75 ml/min. Afterward, 660 μl of 25 wt% glutaraldehyde was added to stabilize the GNPs. Unreacted aldehyde groups were blocked by adding 100 ml of 100 mM glycine solution after 16 h of cross-linking of the GNPs. Then, the suspension was resuspended in deionized water by vortexing after centrifuging at 5000 rpm for 60 min. The suspension was changed to pH 7.0 after three cycles of washing and freeze-drying for 48 h. The GNPs were obtained and sterilized by gamma irradiation.

### Preparation of pTa-GNPs hydrogel scaffold

A 0.13 g freeze-dried GNPs and 1 ml sterile water were filled in the syringe and mixed by repetitive extrusion. The injectable and moldable hydrogel were manufactured within 30 s. The pTa was filled with the injectable hydrogel with the syringe. The morphology of scaffold material was observed using SEM. The scaffold material was frozen and fractured at −80°C, followed by freeze-drying to remove the water of hydrogel. Moreover, to evaluate the surface morphology of the scaffold material, the cross-section was coated with gold to improve the conductivity.

### Isolation, culture, and identification of BMSCs

BMSCs were isolated from the bone marrow of the tibia and femur of a 6-week-old athymic nude mouse. The extracted marrow was centrifuged with the heparin, then mixed and resuspended in the solution containing 10% fetal bovine serum (FBS; Corning, USA), DMEM/F12 (HyClone, USA), and 1% penicillin–streptomycin (HyClone, USA). The culture medium was rinsed using phosphate buffer saline (PBS, HyClone, USA) after 2 days to eliminate the floating cells, and replaced the culture medium.

Osteogenesis and adipogenic differentiation experiments were used to evaluate the differentiation of BMSCs. The BMSCs at the 4th passage were used for induction experiments. The BMSCs at the 3rd passage were harvested and cultured with the growth medium at a density of 5 × 10^4^ cells/well in six-well plates. The osteogenic induction medium is used for BMSCs identification after confluence in the growth medium [22].

Alizarin Red and alkaline phosphatase staining were performed in induction culture on Days 14 and 21, respectively. When staining with Alizarin Red, it removed the medium, rinsed the cell layer with PBS, and fixed in 4% paraformaldehyde solution at room temperature for 20 min. Then, using 0.1% Alizarin Red S to incubate the fixed cells at 37°C for 30 min. For ALP staining, we used the BCIP/NBT ALP Color Development Kit to incubate the fixed cells at 37°C for 30 min. During adipogenesis, the BMSCs at the 3rd passage cultured with the adipogenic differentiation medium at a density of 5 × 10^4^ cells/well in six-well plates. After 3 weeks of culture, the oil red O staining method was used to assess the adipogenic ability of the BMSCs.

### Endothelial cell differentiation and identification

The BMSCs at the 3rd passage were harvested and cultured with the growth medium at a density of 5 × 10^4^ cells/well in six-well plates. Then, the cells were cultured in the induction medium, including 50 ng/ml VEGF (PeproTech, USA) and 5% FBS for 7 days.

*In vitro* analysis of capillary formation was performed using Angiogenesis Assay Kit (Merck Millipore, Germany). One well of a 96-well plate was filled with 50 µl of gel matrix solution and incubated at 37°C for 60 min. Endothelial-differentiated BMSCs with 5 × 10^3^ cell count were suspended in 150 µl induction medium, plated onto the gel matrix, and incubated for 12 h.

Immunofluorescence was performed to detect the expression of CD31, which is an endothelial-specific marker in endothelial differentiated BMSCs. Firstly, the endothelial differentiated BMSCs were fixed by adding 4% paraformaldehyde for 5 min and 0.2% Triton for 30 min to complete membrane permeation. After blocking for 60 min by 10% FBS, the primary antibody rabbit anti-mouse CD31 (1:100 dilution; ab222783, Abcam, UK) was added and incubated overnight at 4°C. Next, the cells were incubated with secondary antibody Alexa Fluor 488-conjugated goat anti-rabbit (1:200 dilution; ab150077, Abcam, UK) after washing with PBS. Finally, nuclei were stained with 4,6-diamidino-2-phenylindole (DAPI), and the fluorescent images were obtained using laser scanning confocal microscopy (Eclipse Ti, Nikon, Japan).

### Flow cytometry of BMSCs and endothelial differentiated BMSCs

A 0.5% bovine serum albumin (BSA) and 2 mmol/l EDTA were added in PBS, and 5 × 10^5^ cells were incubated in 100 μl prepared PBS solution with 5 μl FITC-conjugated anti-CD31 (561813, BD Biosciences, CA), 5 μl FITC-conjugated anti-CD34 (560238, BD Biosciences), 5 μl APC-conjugated anti-CD44 (559250, BD Biosciences), 5 μl FITC-conjugated anti-CD45 (ab25224, Abcam, UK), 5 μl PE-conjugated anti-CD90(ab24904, Abcam, UK) for 20 min, respectively. The negative control was operated with matched isotype immunoglobulin G to replace the antibody. After extensive washing with PBS, the flow cytometer (FACSCanto II, BD Biosciences) was used to analyze the cells fluorescence with FACSDiva software (v7.0, BD Biosciences).

### Cytotoxicity of the pTa by cell counting kit-8

The cell toxicity of the pTa was evaluated using the BMSCs culture. BMSCs of 5.0 × 10^4^/ml concentration at the 3rd passage were seeded in wells of the 24-well plate, respectively, and the sterilized pTa scaffold was added to each well. In the control group, we did not add pTa sample. After 1, 3, 5, and 7 days of culture, pTa scaffold and the medium were removed, and fresh culture medium with 10% cell counting kit-8 reagent (CCK-8; Dojindo, Japan) was added to each well and incubated for 120 min. Then, the medium containing 10% CCK-8 solution was removed from each well of the 96-well plate and was measured by a microplate reader (Epoch 2, Biotek, USA) at 450 nm.

### Characteristics of cell adhesion on pTa *in vitro*

The cell adhesion was evaluated by the direct contact assay. BMSCs were employed to examine the cytocompatibility of pTa. BMSCs with 1 × 10^5^ cells/ml were suspended and seeded on pTa.

On day 7 of co-culture, PBS was used to rinse the samples, and 2% glutaraldehyde was used to fix them for 120 min. Then, the samples were conducted with serial dehydrated and critical point dried. To improve the conductivity, the samples were coated with gold. The surface morphology was observed using SEM.

### Live cell assay

GNPs hydrogel was filled in the well of a 24-well culture plate, and 5 × 10^5^ cells/ml BMSCs were suspended and inoculated onto the surface of GNPs hydrogel. The uniform colonization was assessed by live-cell assay (Molecular Probes, USA) after 1, 3, and 5 days of co-culture. Scaffold material was covered with calcein AM solution (2 μM), and then incubated for 30 min in the dark. The samples were imaged using laser scanning confocal microscopy (Eclipse Ti, Nikon, Japan).

### *In vivo* vasculogenesis experiments

**Figure 2.**
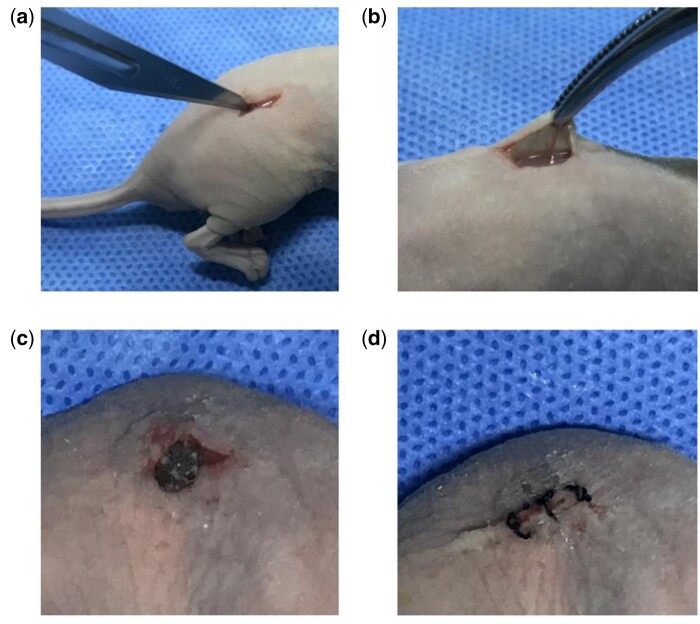
Schematic diagram of animal model. (**a**) Incision of skin tissue on the back. (**b**) Exposure of subcutaneous tissue. (**c**) Implanted composite scaffold material in subcutaneous tissue. (**d**) Incision suture.

**Scheme 1.**
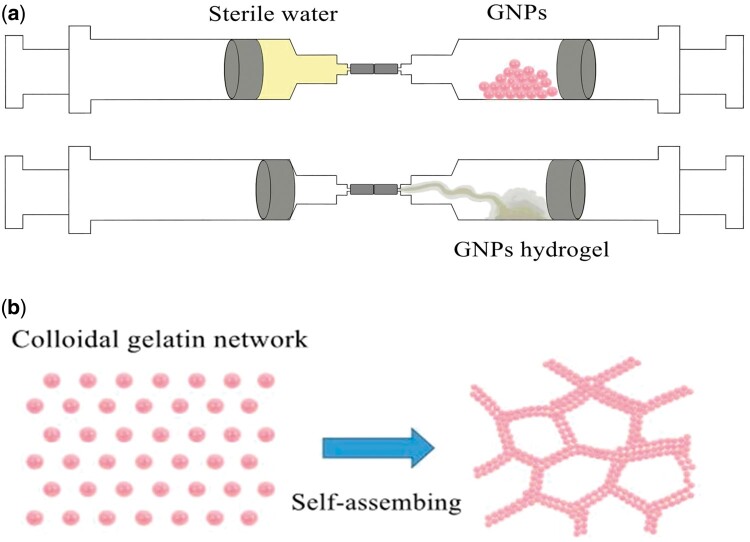
(**a**) Prepare schematic of GNPs hydrogel. (**b**) The formation mechanism of GNPs colloidal network.

**Scheme 2.**
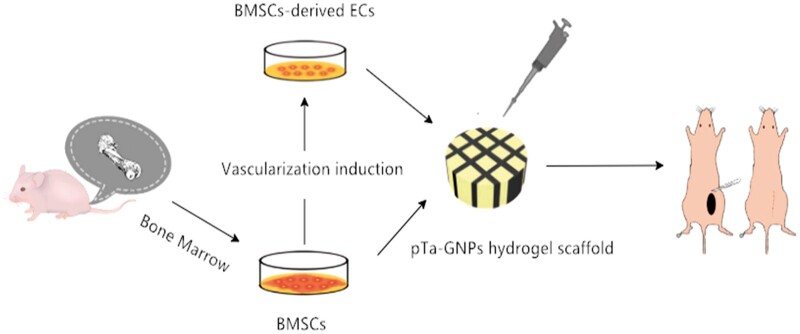
Schematic for constructing vascularized scaffolds in subcutaneous tissue of mouse with pTa-GNPs hydrogel scaffold combined with BMSCs and BMSCs-derived ECs. BMSCs were harvested from mouse tibia and femur. PTa-GNPs hydrogel scaffold were seeded with BMSCs and BMSCs-derived ECs, respectively. Composite material was implanted in subcutaneous tissue of mouse for 4 weeks.

The experimental scheme was carried out according to the China Animal Research Guidelines, and all procedures for animals experiments were approved by the Animal Ethics Committee of Dalian University. All the animals received human care in compliance with the “Guide for Care of Laboratory Animals” which is mentioned by the National Ministry of Science. Twenty 6-week-old male athymic nude mice (30 ± 5 g) were used in this study. Scaffold material pTa was filled with composited GNPs hydrogel (13% w/v). Endothelial-differentiated BMSCs and BMSCs dispersed as individual cells in a 50:50 ratio, or 100% endothelial differentiated BMSCs or 100% BMSCs were prepared for cell-type control experiments. The cells of 5 × 10^5^ cells/ml concentration of different combinations were suspended and seeded on a pTa-GNPs hydrogel scaffold. The control group was designed as a pTa-GNPs hydrogel scaffold without cell implantation (Table 1). After 24 h of co-culture, the composite scaffold material was divided into four groups and was implanted in the subcutaneous tissue of the back by subcutaneous incision. Two composite scaffold materials were implanted per mouse (Fig. 2). Each experimental group contained five mice. The general experimental protocol was shown in Schemes 1 and 2.

**Table 1:** Experimental condition

Experimental condition	Group	Cell types	Media
A	Non	Growth medium + endothelial medium 1:1 v/v
B	BMSC	Growth medium + endothelial medium 1:1 v/v
C	BMSC differentiated to EC	Growth medium + endothelial medium 1:1 v/v
D	BMSC differentiated to EC + BMSC	Growth medium + endothelial medium 1:1 v/v

### Histology

All mice of each group were sacrificed after 28 days of the surgery. The subcutaneous samples were collected and fixed in 10% formaldehyde solution in phosphate buffer (pH 7.4). Then, the specimens were immersed in the graded series of ethanol to dehydrate and embedded in plastic to slice the hard tissue. The sections were sliced to 30 μm thickness for Van Gieson’s stain. Four visual fields of every slice were chosen randomly, and imaged by an optical microscope (BX53, Olympus, Japan).

Microvessel density analysis was used to evaluate the angiogenic ability of samples. The vascular lumenal structures were identified as microvessels, and the amount was counted. Image analysis was used to estimate the area of the hydrogel. To calculate the microvessel density of each sample, we divided the total quantity of microvessels by the total area of hydrogel (expressed as vessels/mm^2^).

### Statistical analysis

All the results were expressed as mean ± standard deviation (SD). The data of groups were analyzed by analysis of variance (ANOVA) followed by post-hoc multiple comparisons using Tukey's test. *P* values < 0.05 were considered to indicate a statistically significant difference.

## Results

### Morphology and mechanical property of pTa scaffolds

**Figure 3.**
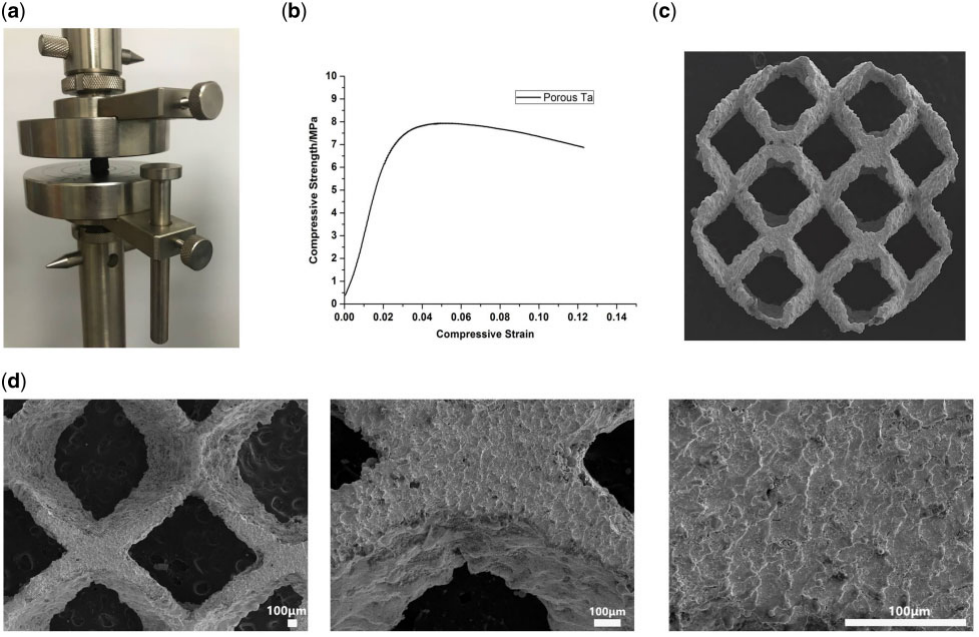
Mechanical properties and surface morphologies of pTa. (**a**) Compression experiment. (**b**) Stress–strain curve. (**c**) Surface morphologies of pTa. (**d**) Flat morphology and the metallic luster of dark gray were shown. These images were magnified ×30, ×100, and ×200, respectively (scale bar = 100μm).

The porosity and pore size of the pTa scaffolds samples were corresponding with the intended design (Table 2). The compression stress and elasticity modulus were shown in Table 3, and the stress–strain curve of pTa was analyzed in Fig. 3b. The surface morphology of pTa was observed by SEM, and the metallic luster of dark grey was visible (Fig. 3c and d).

**Table 2.** Mean porosity, strut size, and pore diameter of pTa scaffolds

Porosity (%)		Strut size (μm)		Pore diameter (μm)	
Design	Calculate	Design	Measure	Design	Measure
88.6	84.3 ± 3.5	430	410 ± 22.5	1300	1265 ± 33.4

**Table 3.** Mean compression stress and elasticity modulus of pTa scaffolds

Compression stress (MPa)	Elasticity modulus (GPa)
8.01 ± 0.24	1.12 ± 0.01

### Characteristic and morphology of pTa-GNPs hydrogel scaffold

**Figure 4.**
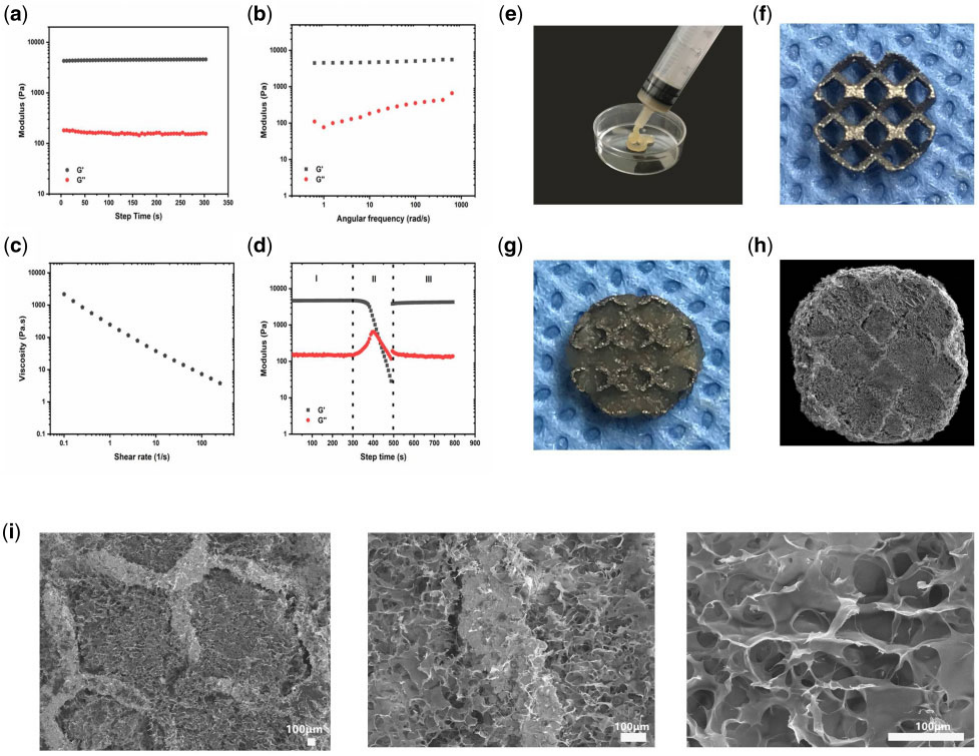
Characteristic and morphology of pTa-GNPs hydrogel scaffold. (**a**) Time dependence of storage (*G*′) and loss(*G*″) modulus of GNPs. (**b**) Frequency dependence of storage (*G*′) and loss (*G*″) modulus of GNPs. (**c**) Viscosity and shear-thinning behavior of GNPs. (**d**) Representative rheological measurement showing the healing behavior of the hydrogels during a cycle of destructive shearing and recovery. (**e**) GNPs’ hydrogels have injectability and adaptability to irregular shapes before completely solidified. (**f** and **g**) The appearance of pTa and pTa-GNPs hydrogel scaffold. (**h** and **i**) The SEM images showed the surface morphology of pTa-GNPs hydrogel scaffold and the representative microstructures were observed. The images were magnified ×30, ×100, and ×300, respectively (scale bar = 100 μm).

GNPs form a rather elastic but also self-healing and shear-thinning colloidal gel on account of the cohesive interactions between globally positively charged but locally amphoteric GNPs [20, 23]. This allows the colloidal gel of GNPs to be extruded through a syringe and easily mixed with other materials to be molded. The rheological properties of GNPs colloidal gel were characterized by rheometer. The modulus of GNPs colloidal gel was time-independent (Fig. 4a). The storage modulus *G*′ value was about 4.5 kPa, and the loss storage *G*″ value was 0.1 kPa. The oscillatory frequency sweep tests indicated the modulus of GNPs was low frequency dependent upon applying constant shear (Fig. 4b). The storage modulus *G*′ value was increased from 4.5 to 5.5 kPa. The viscosity of GNPs colloidal gel was decreasing with the increasing shear rate (Fig. 4c). Moreover, GNPs colloidal gel also revealed a robust degree of mechanical recovery after the network destruction (Fig. 4d). As shown in Fig. 4d, in region I the GNPs colloidal gel indicated higher *G*′ than *G*″. Subsequently, in region II, a destructive shear strain (0.5% for 200 s) led to network destruction and the transformation from a solid gel to a liquid-like material(*G*″ > *G*′). Upon release of the destructive shear, GNPs immediately showed more than 90% of recovery of *G*′ value as relevant to the initial *G*′.

In this study, we prepared the gel with GNPs concentration of 13 w/v% to obtain colloidal gel with high mechanical strength. The high concentration could eliminate the structural changes after shear-induced mixing. GNPs’ hydrogels have injectability and adaptability to irregular shapes before completely solidified (Fig. 4e). The SEM images showed GNPs’ hydrogels and tightly bonded pTa (Fig. 4h). The surface morphology with a pore diameter of 76 ± 15.3 μm and the representative microstructures of GNPs’ hydrogels was observed (Fig. 4i).

### Identification of BMSCs

**Figure 5.**
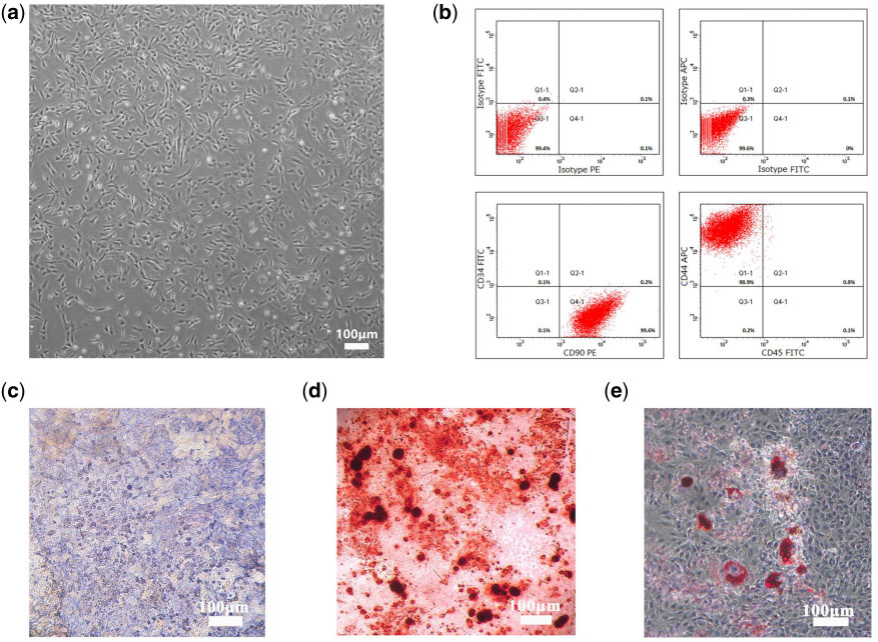
Culture and identification of BMSCs. (**a**) Light microscopic observation of the third-generation BMSCs (scale bar = 100 μm). (**b**) Flow cytometry identification of cell phenotype. CD44 and CD90 were expressed, but CD34a and CD45 were scarcely expressed. (**c**) Alkaline phosphatase was showed after 2 weeks of culture in osteogenic induction medium (scale bar =100 μm). (**d**) After 3 weeks of culture in osteogenic induction medium, Alizarin Red staining showed calcified nodules (scale bar =100 μm). (**e**) The oil red O staining showed the intracellular lipid droplets after 3 weeks of culture in adipogenic induction medium (scale bar = 100 μm).

The typical adherent spindle-like shape of the cells isolated from mice tibia and femur marrow samples was imaged using a microscope (Fig. 5a; CKX41, Olympus, Japan). The BMSC markers CD44 and CD90 were expressed by the extracted cells, but CD34a and CD45 were rarely expressed (Fig. 5b).

The results indicated that the isolated cells from the marrow showed alkaline phosphatase after 2 weeks of culture in an osteogenic induction medium (Fig. 5c). After 3 weeks of culture in osteogenic induction medium, Alizarin Red staining showed calcified nodules, indicating that the isolated cells were at the stage of matrix mineralization (Fig. 5d). The oil red O staining showed the intracellular lipid droplets after 3 weeks of culture in adipogenic induction medium (Fig. 5e).

### Angiogenesis evaluation

**Figure 6.**
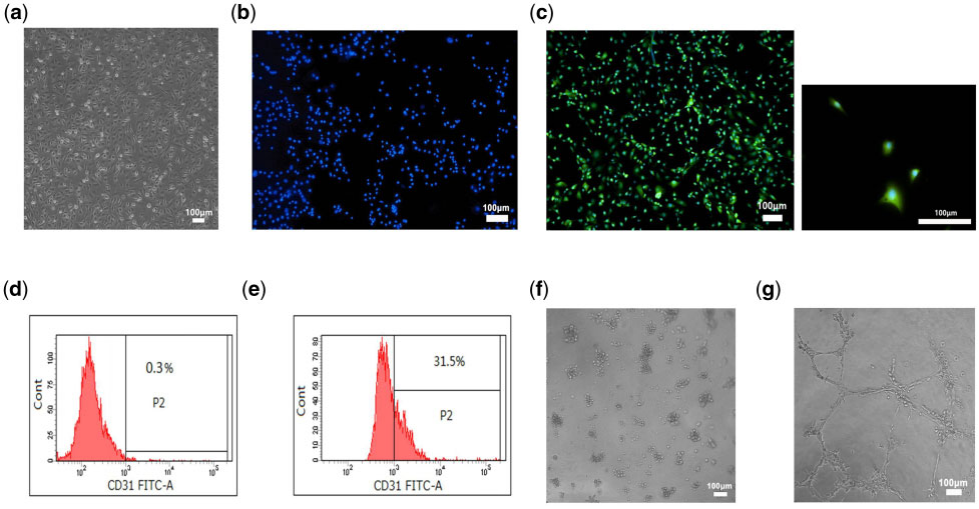
(**a**) Light microscope images of endothelial differentiated BMSCs (scale bar = 100 μm). (**b** and **c**) basal characterization of ECs CD31 expression in BMSCs and endothelial differentiated BMSCs by immunofluorescence. Blue fluorescence signifies DAPI, green indicates CD31 (scale bar = 100 mm). (**d**) Flow cytometry analysis showed the conversion of ECs in BMSCs group is 0.3±0.1% (scale bar = 100 mm). (**e**) In endothelial differentiated BMSCs group, the conversion of ECs is 31.5±1%. (**f**) BMSCs remained around in tube formation assay (scale bar=100 mm). (**g**) Endothelial differentiated BMSCs formed visible tube-like structures on matrigel after 12 h (scale bar = 100 mm).

Compared to the isolated cells, the morphology of bigger and round, cytoplasm shrank and spread with a cobble stone-like appearance was observed after 7 days of culture in the vascularized induction medium (Fig. 6a).

Immunofluorescent staining for CD31 was carried out for the basal characterization of ECs. For CD31 staining, BMSCs showed almost no specific results (Fig. 6b). However, the fluorescence intensity of endothelial differentiated BMSCs increased significantly after 7 days of induction (Fig. 6c).

In the BMSCs group, flow cytometry analysis showed that the percentage of CD31+ cells was 0.3%, and the 31.5% CD31+ cells were observed in the endothelial differentiated BMSCs group (Fig. 6d and e).

The *in vitro* angiogenesis kit was applied to investigate the ability of BMSCs and endothelial differentiated BMSCs to form capillaries in a semi-solid medium. Two kinds of cells were seeded on EC matrix gel solution. After 12 h, nearly 97% of cells were found rounded in the BMSCs group (Fig. 6f). However, differentiated endothelial BMSCs showed visible tube-like structures (Fig. 6g).

### Adhesion and proliferation of BMSCs on pTa *in vitro*

**Figure 7.**
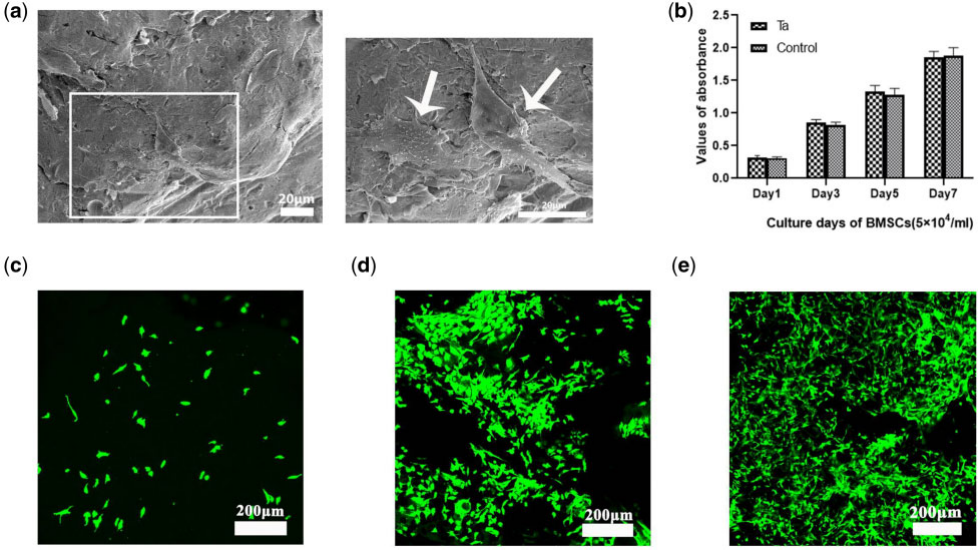
(**a**) SEM morphology of BMSCs attached to pTa scaffolds after 3 days of culture (marked by white arrows; scale bar = 20 μm). (**b**) The CCK-8 assay showed the proliferation of BMSCs after co-culture with pTa for 1, 3, 5, and 7 days. (**c**) Live assay of GNPs hydrogel. On day 1 of co-culture, a small number of BMSCs adhered to GNPs hydrogel. (**d**) On Day 3 of co-culture, the BMSCs adhered nonhomogeneous to GNPs hydrogel, and several cell aggregates were formed. (**e**) The trend of cell proliferation on GNPs hydrogel was evidently on Day 5 of co-culture (scale bar = 200 μm).

The adhesion and morphology of BMSCs were observed by SEM. After 3 days of co-culture, we found the cells attached to the surface of pTa, the cellular morphology displayed the active status and the cellular pseudopodium spread well (Fig. 7a). Our result showed superior cytocompatibility and adhesive of pTa. We expected that the pTa would be gradually covered with proliferation.

The proliferation of cells on pTa was analyzed. Compared with the control group, the statistical results of CCK-8 showed that BMSCs co-cultured with pTa for 1, 3, 5, and 7 days, the proliferation was not inhibited (*P* > 0.05, Fig. 7b). We affirmed that the pTa scaffold had no cytotoxicity to BMSCs.

### Live cell assay

BMSCs were seeded on GNPs hydrogel, and live-cell assay was performed after 1, 3, and 5 days. The bottom scaffold surfaces of each sample were imaged. On day 1 of co-culture, there were a small number of BMSCs adherent to GNPs hydrogel (Fig. 7c). On day 3 of co-culture, the BMSCs adhered nonhomogeneous to GNPs hydrogel, and several cell aggregates were formed (Fig. 7d). The trend of cell proliferation on GNPs hydrogel was observed on day 5 of co-culture (Fig. 7e).

### Histological evaluation

**Figure 8.**
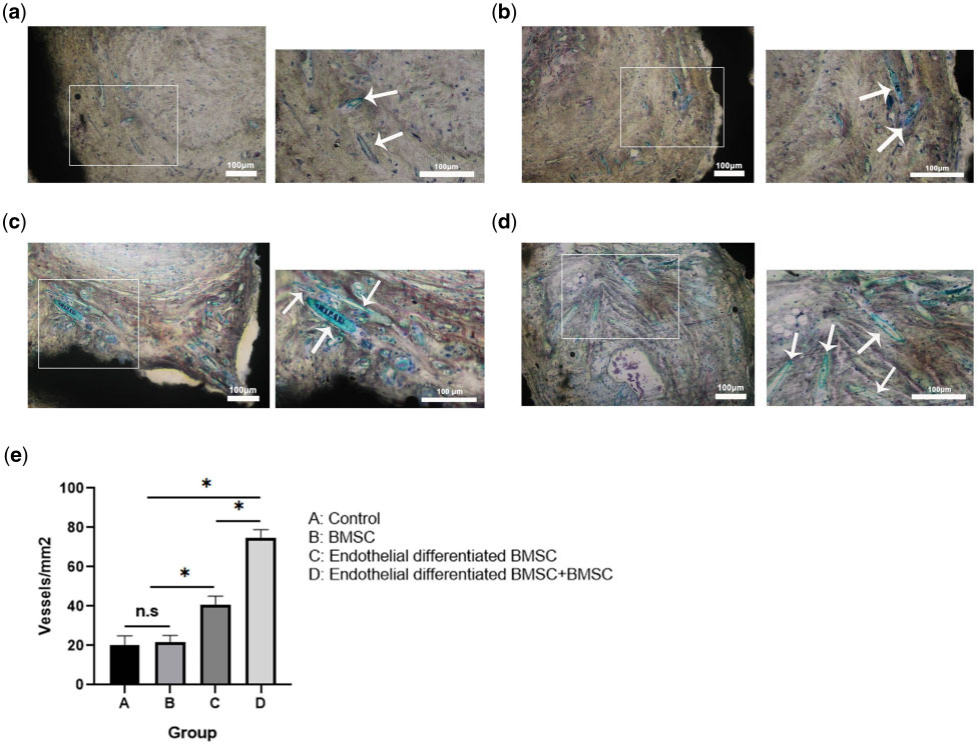
The effect of pTa-GNPs hydrogel scaffold associated with BMSCs and BMSCs-derived ECs on angiogenesis. (**a–d**) Van Gieson’s staining of histological sections of four groups’ implants at 4 weeks post-operation revealed the presence of numerous blood vessels (a: control; b: BMSCs; c: endothelial differentiated BMSCs; d: endothelial differentiated BMSCs+BMSCs). White arrows marked the newly formed blood vessels. The large image was magnified ×100 with a scale bar = 100μm; the small image was magnified ×200 with a scale bar = 100μm (marked by white arrows). (**e**) Histomorphometric analysis of the samples after 28 days of implantation. Quantitative results of vascular lumens determined the microvessel density. Bars represent the mean microvessel density of four groups of implants ± SD. Data were analyzed by analysis of variance (ANOVA) followed by post-hoc multiple comparisons using Tukey's test. *P < 0.05.

After 28 days of implantation, four groups of the subcutaneous samples in mice were taken out. Van Gieson’s staining results showed the difference in the degree of vascularization *in vivo* (Fig. 8a–d). Quantitative results of vascular lumens determined the microvessel density, and the density of the four groups is shown in Fig. 8e. The pair comparison of differences among B, C, and D groups was statistically significant (*P* < 0.05). When compared with group A, group B had similar microvessel density without significant difference (*P* > 0.05), whereas both of groups C and D had significantly higher microvessel density than group A (*P* < 0.05).

## Discussion

The application of tissue vascularization is the major challenge for the clinical transformation of TE. This study aimed to construct artificial cellular scaffolds with mechanical strength and appropriate physiological microenvironment to mimic the microstructure of natural tissue for pTa application in vascularized TE.

In summary, we developed a 3D pTa-GNPs hydrogel scaffold, which is biocompatible with the host and exhibits biomechanical behavior. Furthermore, we explored the methods of endothelial induction of BMSCs and 3D co-culture with the scaffold. Then, the angiogenic properties of pTa-GNPs hydrogel scaffold combined with BMSCs-derived ECs *in vivo* models were assessed.

The 3D printing technology has developed rapidly in recent years and is widely used in TE. It could offer superior flexibility in manufacturing 3D samples with complex geometries [24]. Therefore, TE is constantly developing novel scaffold materials to improve the corresponding mechanical properties. In addition, 3D printing can directly use biomaterial powder to manufacture implants with complex structures to substitute tissue and achieve the desired effect through precise structural design [25, 26]. Given its excellent tissue compatibility, high porosity, appropriate surface friction coefficient, and low elastic modulus, pTa has been recognized as an ideal orthopedic implant material. Moreover, with the continuous progress of 3D printing technology, pTa has a broad scope of applicability [27]. Wauthle *et al*. used SLM technology to manufacture highly pTa with entirely interconnected open pores, which approximated mechanical properties of human bone, and the pTa has excellent osteoconductive properties, normalized fatigue strength, and high strength ductility [28]. Wang *et al*. aimed to compared with traditional porous Ti implant, the Ti and Ta samples were manufactured with the same pore structure by computer design, and then SLM was used to produce the samples. It suggested that pTa has the same promotion effect on bone fixation and equal biological performance [29]. Consistent with our previous studies [30], most pTa's studies as the biomaterial was conducted on the compatibility and mechanical support. The key problem for bone regeneration *in vivo* is a vascular deficiency in the engineered structure [1]. However, pTa has no microstructure to mimic the ECM environment, lacking the characteristics to promote vascularization. Therefore, we used SLM technology to manufacture pTa with a specific structure and add suitable filling materials in the macropores of pTa to construct the suitable microstructure.

The hydrogel with local adaptability and long-term volume stability usually formed by reversible interactions (such as electrostatic interactions, hydrogen bonds, hydrophilic/hydrophobic interactions, and host–guest interactions) has become an attractive biomaterial [31]. According to our previous studies [32], the GNPs hydrogel exhibited a network mechanism, and the self-assembled colloidal network could disperse the biological stress. The scaffold of gelatin colloidal gels contains a porous but interconnected particulate network dispersed in the aqueous solvent. In TE, nanostructured colloidal gelatin gels are excellent carriers that can promote the programmed and sustained release of multiple proteins and also support cells to adhere, spread, and proliferate *in vitro*. However, due to weak reversible bonds' inherent characteristics, these adaptable hydrogels show insufficient mechanical strength, limiting their wide application as biomaterials *in vivo*.

Therefore, we manufactured a new type of 3D scaffold material combined with pTa and GNPs hydrogel, to enable the material to possess certain mechanical strength for cell and tissue regeneration and provide a physiological relevant microenvironment. We designed the parameter of the pTa as 1300 μm (pore size), 430 μm (strut size), and 88.6% (porosity) in this study. Moreover, mechanical testing showed that the compressive strength and the elastic modulus were 8.01 MPa and 1.12 GPa, respectively. It can optimize mechanical properties and provide enough space for filling materials to promote vessel formation and growth. Meanwhile, SLM technology was used to control the structure of pTa precisely. The rheological characterization confirmed that GNPs colloidal gel was a typical viscoelastic gel-like material. The fantastic combination of pTa scaffold and gels was attributed to the viscoplasticity of the gel. The shear-thinning and self-healing behavior of GNPs was observed. Due to the self-healing behavior, the GNPs colloidal gel could be injected through the syringes needle. The property of the material also means that it was residing in the implantation site.

The scaffold material should maintain the interconnected and continuous network of pores, which is the critical factor for uniform cell seeding, promoting nutrient transportation and waste removal, and thus improve the vascularization performance of the scaffold material [33]. The surface morphology of GNPs and the representative microstructures were observed using SEM. It showed a porous structure with a pore diameter of 76 ± 15.3 μm. The pore size of the scaffold material affects cell adhesion and vascular growth [34]. Previous studies have shown that when the pore size of the scaffold is larger than 50 μm, it can achieve the diffusion of nutrients, oxygen, and the excretion of metabolites, and porous material with a pore size of 100–200μm is conducive to the vascularization [35, 36]. According to our previous studies [20, 37], GNPs colloidal gel had superior performance combining mechanical properties and self-healing capacity. Moreover, it is developed by elaborative control of particle assembly, a basic understanding of the formation mechanism and accurate adjustment of the structure and composition of the gel network. The organic and inorganic colloidal building blocks were composed of amphoteric soft GNPs and negatively charged hard silica nanoparticles, respectively. When the net charge of the GNPs alters from negative to positive, the self-assembly reaction between the oppositely charged GNPs and silica is triggered. Although long-range attractive electrostatic interactions caused the formation of the gel network, the subsequent formation of additional short-range particle interactions (such as hydrogen bonds and van der Waals) exceeded the repulsive electrostatic forces, maintaining the integrity of the gel network.

The biocompatibility of the implant can be assessed by the interaction between implants and BMSCs [38]. Cell adhesion and proliferation was performed to assess interactions of material and BMSCs. The interaction was assessed by SEM cell morphology, CCK-8 kit, and live-cell assay. Our results showed that the pTa and GNPs’ hydrogels have a superior biocompatible for cell adhesion, viability, and proliferation.

Previous studies have shown that it is possible to establish the microvascular network using mature ECs derived from vascular tissue. However, the clinical application of ECs taken from autologous vascular tissue is limited because there is no program to get sufficient cells [2]. MSCs have great potential in regenerative medicine and have certain application value in complex TE [14]. There are many studies that report BMSCs for treating bone and cartilage defects, as well as the damaged myocardium after acute myocardial infarction [39]. Studies have confirmed that BMSCs can differentiate into different kinds of connective tissues, including osteocyte, chondrocyte, and adipocyte, and also can differentiate into ECs and vascular smooth muscle cells [40]. In this study, the expression patterns and the multiple lineage differentiation capacities of BMSCs were assessed by flow cytometry and differentiation experiment, respectively. Moreover, the results showed that our extracted cells were comprised identified BMSCs, which have the potential of multiple differentiation. We induced BMSCs into EC-like cells successfully in the induction medium. The expression of EC surface marker CD31 was detected by flow cytometry. The endothelial differentiated BMSCs formed the visible tube-like structures that showed the functional properties of ECs.

BMSCs cannot differentiate into blood vessels spontaneously *in vivo*; however, recent evidence suggested that it can differentiate into perivascular cells *in vivo* to promote angiogenesis. Therefore, BMSCs play the role of pericytes in the formation of new blood vessels, and can stabilize and maintain the development of the vascular system [41]. Furthermore, there is a heterogeneous interaction between BMSCs and ECs, providing survival advantages for ECs and perivascular cells [42]. The vascularization performance of the material can be effectively evaluated by implanting it on the back of mice [43, 44]. In this study, BMSCs-derived ECs and BMSCs were seeded on scaffold materials to participate in angiogenesis. Subsequently, we compared the angiogenesis property of the scaffold material on the back of nude mice. After 28 days of implantation, the histomorphometry statistics of the neovascularization in terms of density suggested that group C (40 ± 8 vessels/mm^2^) was significantly superior compared to group A (20 ± 9 vessels/mm^2^) and B (21 ± 7 vessels/mm^2^). These results indicate that during vascularization *in vivo*, BMSCs-derived ECs had a significant impact and the cells have great potential application in the research of regenerative medicine. Compare to group C (40 ± 8 vessels/mm^2^), the values of group D (75 ± 6 vessels/mm^2^) suggested the interaction between BMSCs-derived ECs and BMSCs that could form the vessel in the composite scaffold materials, and the co-culture of two types of cells *in vivo* could affect the maturation and stability of the vascular network (Fig. 7). Meanwhile, group B (21 ± 7 vessels/mm^2^) was similar to group A (20 ± 9 vessels/mm^2^), suggesting that BMSCs played a synergistic role in the process of vascularization rather than a decisive. In addition, these results demonstrated that the pTa-GNPs hydrogel scaffold was biologically active *in vivo* and promoted the formation of a capillary-like network *in vivo*. Due to the insufficient mechanical strength, GNPs hydrogel cannot maintain its original shape after subcutaneous implantation. Therefore, the GNPs hydrogel group was not set in this study.

Based on our previous studies [23, 45], the colloidal hydrogel has some specific structure and performance such as inherent porous matrix, the large specific surface area of gelatin, electrostatic and hydrophobic interactions with a strong affinity for proteins. Moreover, it could continuously release biomolecules. In this study, GNPs’ hydrogels probably provided the physiologically concerned microenvironment for tissue regeneration and played a role as absorbent and prolonged the release of biologically active substances; thereby, it could induce EC generation and angiogenesis. In addition, though cells were randomly implanted, the live-cell assay showed the BMSCs adhered nonhomogeneous, tissue sections showed the newly formed blood vessels were unevenly distributed. The influence of the mechanical strength and microporous structure of GNPs on cellular immunity and migration might affect this phenomenon; however, it has not been explored in this study.

In recent years, 3D bioprinting technology is rapidly developing. Among them, extrusion-based systems, inkjet printing systems, and laser-based technologies have been developed to accurately distribute cells in 3D structures [46, 47]. The precise distribution of human umbilical vein EC and smooth muscle cell with biolaser printing technology can control the formation of the vessel network, as well as the size and shape of the lumen [48]. Contrarily, using thermal inkjet printer manufacturing technology, a thrombin solution containing ECs was deposited on the fibrinogen matrix to create a 3D tubular microvascular structure [49]. Instead of using a computer-controlled deposition system, we randomly implant cells in scaffold material to allow the cells to organize themselves. However, BMSCs-derived ECs were not labeled and tracked in this study. Therefore, the location and number of these cells in the neovascularization could not be determined.

## Conclusion

In summary, novel, integrated 3D scaffold materials were designed with biocompatibility, biomechanical and angiogenic properties, and an approach for TE to construct vascular engineered tissue. We used the SLM technology to manufacture pTa and filled the macropores of pTa with GNPs hydrogel. The pTa and GNPs hydrogel had no inhibitory effect on the proliferation of BMSCs *in vitro*. The BMSCs-derived ECs were implanted on pTa-GNPs hydrogel scaffold, and they could promote the formation of a capillary-like network *in vivo*. Therefore, endothelial differentiated BMSCs played a significant role in TE. This study might provide a basis for applying pTa in bone regeneration; meanwhile, tissue-engineered vascular grafts based on autologous BMSCs deserved further investigation. In bone development, vascularization and mineralization coincided and could make bone the highly vascularized tissue. This work demonstrated the potential to realize the vascularization of TE bone in further studies.

## Funding

This work was supported by Postdoctoral Science Foundation of China (No. 194012), National Natural Science Foundation of China (No. 82172398), Science & Technological Convenience Foundation of Dalian (No. 2020JJ27SN076), Doctoral Research Starting Foundation of Affiliated Zhongshan Hospital of Dalian University (No. DLDXZSYY-BK201809).

*Conflict of interest statement*. None declared.
